# Patient adherence in orthodontics: a protocol for a scoping review

**DOI:** 10.1038/s41405-024-00249-w

**Published:** 2024-07-30

**Authors:** R. M. van der Bie, A. Bos, J. J. M. Bruers, R. E. G. Jonkman

**Affiliations:** 1 Department of Orthodontics, Academic Centre for Dentistry Amsterdam (ACTA), University of Amsterdam and Vrije Universiteit, Amsterdam, The Netherlands; 2 Department of Oral Public Health, Academic Centre for Dentistry Amsterdam (ACTA), University of Amsterdam and Vrije Universiteit, Amsterdam, The Netherlands

**Keywords:** Dentistry, Orthodontics

## Abstract

**Background:**

Patient adherence is a key factor in achieving orthodontic success. While in recent years there have been changes in orthodontic healthcare, no recent comprehensive reviews regarding adherence in orthodontics are available. Therefore, the aim of this planned scoping review is to systematically map the available literature regarding patient adherence in orthodontics to identify factors associated with patient adherence and to investigate if there are knowledge gaps in the available literature.

**Methods/design:**

This protocol was drafted according to guidelines of the Preferred Reporting Items for Systematic Reviews and Meta-analysis Protocols (PRISMA-P) statement and the PRISMA extension for Scoping Reviews (PRISMA-ScR). For the methods Arksey and O’Malley’s framework and the Reviewer’s Manual of the Joanna Briggs Institute for conducting scoping reviews were consulted. The inclusion criteria for this scoping review are studies of all designs assessing any form of adherence in orthodontics, published in English from 2006 onwards. The exclusion criteria are studies investigating adherence in the following patients: those with an intellectual or physical disability that could affect their ability to coincide with their therapist’s recommendations and advice, those with oral cleft and craniofacial conditions, and those solely treated for obstructive sleep apnoea. Case reports and studies published in non peer reviewed journals will also be excluded. The following electronic databases will be searched: Embase, PubMed, and Web of Science Core Collection. The following key terms will be used in the search strategies: ‘treatment adherence and compliance’, and ‘orthodontics’. Multiple reviewers will independently screen the results and perform the data charting process. A narrative description will be provided for the analysis of the included studies. The results will be categorized into multiple topics based on recommendations by previous studies into patient adherence. Identified knowledge gaps will be reported and recommendations for future research will be suggested.

**Discussion:**

No systematic review has previously assessed this exact topic. Because of the broad-spectrum research questions and the expected widely scattered literature a scoping review approach was chosen over a systematic review approach. The Academic Centre for Dentistry Amsterdam (ACTA) has been conducting research in patient adherence in orthodontics up to 2006 and therefore only studies published from 2006 onwards will be researched in this review. Identifying knowledge gaps and summarizing and disseminating research findings on this topic is important for every dental professional performing orthodontic treatment. This protocol is registered in the Open Science Framework: https://osf.io/ec6qd

## Introduction

Patient adherence is regarded as a key factor in achieving orthodontic treatment success [1–3]. Poor adherence may result in less satisfactory treatment outcomes, deleterious effects, prolonged orthodontic treatment, and relapse after treatment [1, 3]. The demand for orthodontic treatment is substantial and in recent years there have been changes in orthodontic healthcare. There has been an increase in the number of adult patients treated and a rise in demand for more aesthetic forms of orthodontic treatment has been reported [4]. Advances in techniques have let to innovations of orthodontic appliances, such as the development of clear aligner therapy, which has gained significant popularity [5, 6]. Also the development of so called ‘non-compliance’ appliances as implant supported appliances [7] can be mentioned.

However, our initial searches did not identify any recent comprehensive reviews regarding patient adherence in orthodontics. Therefore, this manuscript presents the protocol of a planned scoping review. The aims are to systematically search, explore and map the available literature regarding multiple aspects of patient adherence in orthodontics, to identify factors associated with patient adherence, and to investigate if there are knowledge gaps in the available literature. We chose a scoping review approach over a systematic review approach because of the broad-spectrum topic and the expected widely scattered literature. This review explores the literature regarding patient adherence during both active orthodontic treatment as well as the retention phase. Regarding adherence in the active orthodontic treatment phase this study explores the literature of patient adherence in multiple treatment methods and during multiple phases of active orthodontic treatment. The results of this study may be used to conduct further research to explore the fields in which identified knowledge gaps exist.

While in healthcare patient adherence is usually defined as ‘the extent to which a person’s behaviour coincides with medical or health advice’[8], there is no precise definition of patient adherence in orthodontics. Since both the terms ‘adherence’ and ‘compliance’ are commonly used in dentistry [9] these terms will be used interchangeably in this review.

Based on the objective of this scoping review, we have formulated the following research questions:

- How is patient adherence defined in the field of orthodontics?
- What is known about the level of patient adherence in orthodontics?
- How is this level assessed and what can be said about the validity and reliability of these methods?
- What are the factors that influence patient adherence in orthodontics?
- What is known about the promotion of patient adherence in orthodontics?

The Academic Centre for Dentistry Amsterdam (ACTA) has been conducting research in patient adherence in orthodontics up to 2006 [10] and therefore only studies published from 2006 onwards will be researched in this review.

## Materials and methods

### Reporting and conducting of the scoping review

**Fig. 1:**
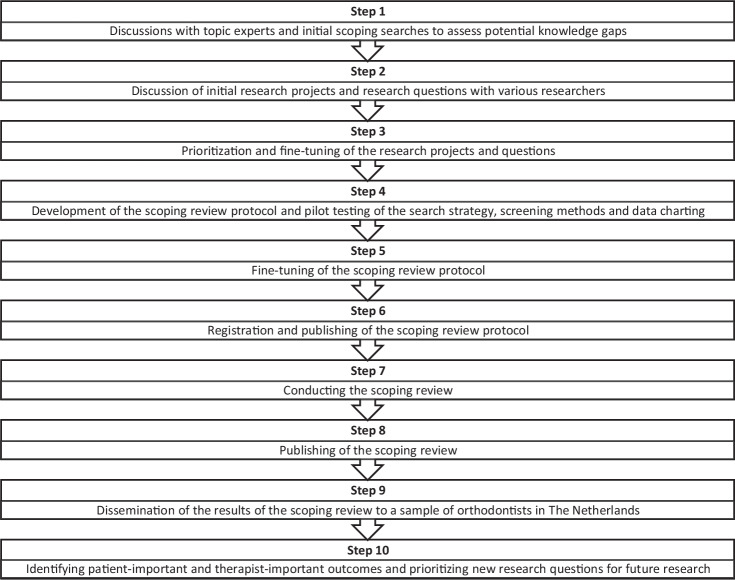
Flow diagram of the current and future research projects.

The protocol for this scoping review was drafted according to guidelines of the Preferred Reporting Items for Systematic Reviews and Meta-analysis Protocols (PRISMA-P) statement [11, 12] (Additional file 1a) and the PRISMA extension for Scoping Reviews (PRISMA-ScR) [13] (Additional file 1b). For the methods of this review we consulted Arksey and O’Malley’s framework [14] and the Reviewer’s Manual of the Joanna Briggs Institute (JBI) for conducting scoping reviews [15] as well. We registered our protocol a priori in the registries of the Open Science Framework (10.17605/OSF.IO/EC6QD). Ethical approval for this scoping review protocol was granted by the Ethical Committee of ACTA on 11 February 2022. Our planned and future research projects are reported in a flow diagram (Fig. 1).

### Eligibility criteria

The following selection criteria will be applied:

- Sources of evidence: studies of all designs with the primary aim of investigating any form of patient adherence in orthodontics will be included, with the exception of case reports and studies investigating adherence in the following patients: those with an intellectual or physical disability that could affect their ability to coincide with their therapist’s recommendations and advice, those with oral cleft and craniofacial conditions, and those treated solely for obstructive sleep apnoea. Patients with oral cleft and craniofacial conditions are excluded because of the higher orthodontic burden for this group of patients [16]. Patients solely treated for obstructive sleep apnoea are excluded because of the difference in treatment need and used appliances for this group of patients. Research in adherence for these groups of patients should be reported in separate reviews.
- Publication type: peer reviewed manuscripts only. No grey literature sources will be included.
- Publication date: eligible studies published from 2006 onwards will be included. The ACTA has been exploring patient compliance in orthodontics up to 2006 [10].
- Publication language restrictions: only eligible studies published in English will be included.

### Information sources and search strategy

The information sources and key terms have been selected in consultation with a medical information specialist working at the Vrije Universiteit (VU) Amsterdam medical university library. The following electronic databases will be searched: Embase, PubMed, and Web of Science Core Collection. The following key terms, including synonyms and subheadings of the MeSH terms, will be used in the search strategies: ‘treatment adherence and compliance’ and ‘orthodontics’. Two reviewers (RB and RJ) pilot tested these strategies. A draft search strategy for PubMed is presented in Additional file 2. The draft search resulted into 3201 results. Since our selection criteria require a publication date from 2006 onwards, we decided to apply this search restriction immediately to make the selection of eligible studies more convenient. The search will be performed by the medical information specialist. Citation tracking and searching of reference lists of included eligible studies will be performed to identify additional eligible relevant research.

### Selection of sources of evidence

The results will be screened independently by multiple reviewers (RB and RJ) in two stages. In the first stage, results will be screened based on the studies’ title, abstract and keywords to identify eligible publications using a conducted first stage screening form (Additional file 3). Rayyan, a free web and mobile application will be used for this first stage. In the second stage, after identification of relevant studies, full-text articles will be obtained and a final selection of studies to be included in our scoping review will be made based on our eligibility criteria using a conducted second stage screening form (Additional file 4). EndNote will be used as the reference management software program. Two reviewers (RB and RJ) pilot tested the two screening stages using 50 randomly selected studies from the 3201 results of the draft search strategy and fine-tuned the screening forms. Potential disagreements between the two reviewers during the two-stage screening will be resolved by rereading of the pertinent studies. Persisting disagreements will be resolved by independent validation by a third reviewer (AB or JB) to either reach consensus or to cast a decisive vote for selection. All steps of the screening and selection process will be presented in a PRISMA flow diagram [11].

### Data charting process

For data extraction, a data charting form (Additional file 5) was conducted using the data extraction template and guidance for scoping reviews of the Joanna Briggs Institute [15] and the checklists of the Enhancing the Quality and Transparency Of health Research Network (EQUATOR Network). Two reviewers (RB and RJ) pilot tested the data charting form. The data charting process will be performed by the two reviewers who selected the sources of evidence (RB and RJ). Potential disagreements between the two reviewers will be resolved by rereading of the pertinent studies. Persisting disagreements will be resolved by independent validation by a third reviewer (AB or JB) to reach consensus on the data to be extracted.

### Quality assessment and risk of bias

Quality appraisals and risk of bias assessments are optional when conducting a scoping review [13, 14] and are typically not performed. Due to the expectation that the eligible studies will include various study designs and will have a lack of reported quantitative outcome measures, we will not perform a quality appraisal or risk of bias assessment or perform any quantitative analyses of the results.

### Synthesis of the results

**Fig. 2:**
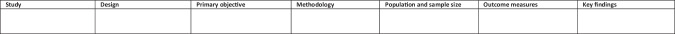
Draft table to summarize the characteristics and key findings of the included studies.

A narrative description will be provided for the analysis of the included studies containing the year of publication, design of the study, objective of the study, methodology of the study, population and sample size of the study, outcome measures of the study (if described), and any key findings that relate to our research questions. Tables will be created to report the characteristics and results of the eligible studies. A draft table to summarize the characteristics and key findings of the included studies is presented in Fig. 2.

The results will be categorized into the following topics, partially based on recommendations by previous research into compliance in orthodontics [2]:

- What is known about the definition of patient adherence in orthodontics?
- What is known about the effects of patient adherence on orthodontic treatment outcomes?
- What is known about the methods of measuring patient adherence in orthodontics?
- What is known about the degree of patient adherence during active orthodontic treatment, taking into account different types of appliances and different stages of treatment?
- What is known about the degree of patient adherence during the orthodontic retention phase?
- What is known about factors to influence patient adherence and methods to promote patient adherence in orthodontics?

Identified knowledge gaps will be reported and recommendations for future research will be suggested.

## Discussion

The proposed scoping review will systematically map the available literature regarding patient adherence in orthodontics. We will assess and synthesize the literature on this research topic, identify knowledge gaps within the available literature, consider the clinical implications, and provide recommendations for future research. The rationale for only including studies published from 2006 onwards is that the ACTA has been conducting research in patient adherence in orthodontics up to 2006 [10]. The rationale for excluding studies investigating patient adherence in orthodontics in patients with oral cleft and craniofacial conditions is that this patient group has a high orthodontic burden when compared to patients without these conditions. This patient group is generally treated in specialized teams and has a longer duration of treatment [16, 17]. Research in adherence for this patient group should therefore be reported in a separate review. Any changes made to this protocol when conducting the scoping review will be reported in the final manuscript and in the Open Science Framework. We will present the type and timing of these changes as well as the rationale and the potential consequences of these modifications.

### Strengths and limitations

No systematic review has previously assessed this exact topic. Because of the broad-spectrum research questions and the expected widely scattered literature a scoping review approach was chosen over a systematic review approach. The strengths of this scoping review include the broad spectrum of information sources, a research team consisting of topic experts and information scientists, pilot-tested research methods, and peer reviewed search strategies. Scoping reviews have some limitations compared to systematic reviews, for example registration of the review protocol is not possible in PROSPERO, there is no mandatory risk of bias assessment, quality assessment or critical appraisal, and no quantitative synthesis [18]. We addressed some of these limitations by registering our protocol a priori in the Open Science Framework.

### Importance and beneficiaries

Conducting of a scoping review is important to identify the need to conduct research in a field when little or no primary studies are identified. Identifying knowledge gaps and summarizing and disseminating research findings on this topic is important for every dental professional performing orthodontic treatment. We will disseminate our findings to a sample of orthodontists in The Netherlands to identify patient-important and therapist-important outcomes and to prioritize new research questions for future research.

### Supplementary information

Additional file 1a

Additional file 1b

Additional file 2

Additional file 3

Additional file 4

Additional file 5

## Data Availability

All data generated or analysed for this research study are reported in this manuscript and the supplementary files.

## References

[1] Al-Moghrabi D, Salazar FC, Pandis N, Fleming PS. Compliance with removable orthodontic appliances and adjuncts: A systematic review and meta-analysis. Am J Orthod Dentofac Orthop. 2017;152:17–32. 10.1016/j.ajodo.2017.03.019 28651764

[2] Bos A, Hoogstraten J, Prahl-Andersen B. Towards a comprehensive model for the study of compliance in orthodontics. Eur J Orthod. 2005;27:296–301. 15947231 10.1093/ejo/cji003

[3] Fleming PS, Scott P, DiBiase AT. Compliance: getting the most from your orthodontic patients. Dent Update. 2007;34:565–6. 18087927 10.12968/denu.2007.34.9.565

[4] Rosvall MD, Fields HW, Ziuchkovski J, Rosenstiel SF, Johnston WM. Attractiveness, acceptability, and value of orthodontic appliances. Am J Orthod Dentofac Orthop. 2009;135:276.e1–12. 10.1016/j.ajodo.2008.07.011 19268820

[5] Alansari RA, Faydhi DA, Ashour BS, Alsaggaf DH, Shuman MT, Ghoneim SH, et al. Adult Perceptions of Different Orthodontic Appliances. Patient Prefer Adherence. 2019;13:2119–28. 31853175 10.2147/PPA.S234449 PMC6916694

[6] Robertson L, Kaur H, Fagundes NCF, Romanyk D, Major P, Flores Mir C. Effectiveness of clear aligner therapy for orthodontic treatment: A systematic review. Orthod Craniofac Res. 2020;23:133–42. 31651082 10.1111/ocr.12353

[7] Bellini-Pereira SA, Pupulim DC, Aliaga-Del Castillo A, Henriques JFC, Janson G. Time of maxillary molar distalization with non-compliance intraoral distalizing appliances: a meta-analysis. Eur J Orthod. 2019;41:652–60. 31107942 10.1093/ejo/cjz030

[8] Haynes RB, Dantes R. Patient compliance and the conduct and interpretation of therapeutic trials. Control Clin Trials. 1987;8:12–9. 3568692 10.1016/0197-2456(87)90021-3

[9] Asimakopoulou K, Daly B. Adherence in dental settings. Dent Update. 2009;36:626–30. 20166379 10.12968/denu.2009.36.10.626

[10] Bos, A *Compliance in Orthodontics*. (2006) [Thesis, fully internal, Universiteit van Amsterdam].

[11] Page MJ, McKenzie JE, Bossuyt PM, Boutron I, Hoffmann TC, Mulrow CD, et al. The PRISMA 2020 statement: an updated guideline for reporting systematic reviews. BMJ. 2021;372:n71. 33782057 10.1136/bmj.n71 PMC8005924

[12] Page MJ, Moher D, Bossuyt PM, Boutron I, Hoffmann TC, Mulrow CD, et al. PRISMA 2020 explanation and elaboration: updated guidance and exemplars for reporting systematic reviews. BMJ. 2021;372:n160. 33781993 10.1136/bmj.n160 PMC8005925

[13] Tricco AC, Lillie E, Zarin W, O’Brien KK, Colquhoun H, Levac D, et al. PRISMA Extension for Scoping Reviews (PRISMA-ScR): Checklist and Explanation. Ann Intern Med. 2018;169:467–73. 30178033 10.7326/M18-0850

[14] Arksey H, O’Malley L. Scoping studies: towards a methodological framework. Int J Soc Res Methodol. 2005;8:19–32. 10.1080/1364557032000119616

[15] Peters MDJ, Godfrey C, McInerney P, Munn Z, Tricco AC, Khalil, H Chapter 11: Scoping Reviews (2020 version). Aromataris E, Munn Z, editors. *JBI Manual for Evidence Synthesis*. JBI; 2020. Available from https://synthesismanual.jbi.global. 10.46658/JBIMES-20-12 (date last accessed 19 March 2024).

[16] Roguzińska S, Pelc A, Mikulewicz M. Orthodontic-care burden for patients with unilateral and bilateral cleft lip and palate. Dent Med Probl. 2020;57:411–6. 33448166 10.17219/dmp/125874

[17] Hameed O, Amin N, Haria P, Patel B, Hay N. Orthodontic burden of care for patients with a cleft lip and/or palate. J Orthod. 2019;46:63–7. 31056071 10.1177/1465312518823010

[18] Munn Z, Peters MDJ, Stern C, Tufanaru C, McArthur A, Aromataris E. Systematic review or scoping review? Guidance for authors when choosing between a systematic or scoping review approach. BMC Med Res Methodol. 2018;18:143. 30453902 10.1186/s12874-018-0611-x PMC6245623

