# Supplementary material for: Patient adherence in orthodontics: a protocol for a scoping review
Source: BDJ Open. 2024 Jul 30;10:62. doi: 10.1038/s41405-024-00249-w (PMC11289492; doi:10.1038/s41405-024-00249-w)
Supplement: Supplementary file 3 — Additional file 2 [file 41405_2024_249_MOESM3_ESM.pdf]

CONTENTS

1. PubMed RL20211126 (3201)....1
1.1. History and Search Details....2

1. PUBMED RL20211126 (3201)

Search blocks generated by RL using (synonyms of) entry terms for

“Treatment Adherence and Compliance"[Mesh] and "Orthodontics"[Mesh]

and their subheadings.

Patient Compliance

"Treatment Adherence and Compliance"[Mesh] OR ((“patient*”[tiab] OR “Client*”[tiab] OR “Treatment*”[tiab] OR “Therapeutic*”[tiab] OR “medication*”[tiab]) AND (“Complian*"[tiab] OR “Adheren*”[tiab] OR “Noncomplian*”[tiab] OR “Nonadheren*”[tiab] OR “cooperati*”[tiab] OR “noncooperati*”[tiab] OR “acceptance*”[tiab] OR “nonaccept*”[tiab] OR “persistence*”[tiab] OR “nonpersistence*”[tiab] OR “no show*”[tiab]))

Orthodontics

"Orthodontics"[Mesh] OR "Orthodont*"[tiab] OR “Dental Marginal Adaptation*”[tiab] OR “Dental internal Adaptation*”[tiab] OR “Dental internal fit*”[tiab] OR “Mandibular Advance*”[tiab] OR “Fixed Functional Appliance*”[tiab] OR “Retainer*”[tiab] OR “Fixed Appliance*”[tiab] OR “Herbst Appliance*”[tiab] OR “Frankel Function*”[tiab] OR “Bimler Appliance*”[tiab] OR “Kinetor*”[tiab] OR “Dental Brace*”[tiab] OR “Clear Aligner*”[tiab] OR “Invisalign”[tiab] OR “transparent Aligner*”[tiab] OR “invisible Aligner*”[tiab] OR “crown lengthen*”[tiab] OR “Occlusal Adjustment*”[tiab] OR “Occlusal Equilibration*”[tiab] OR “Forced Eruption*”[tiab] OR “Tooth Extrusion*”[tiab] OR “Palatal Expansion Techn*”[tiab] OR “Maxillary Expansion*”[tiab] OR “Tooth Movement*”[tiab] OR “Tooth Uprighting*”[tiab] OR “Tooth Intrusion*”[tiab] OR “Tooth

Depression*”[tiab] OR “serial extraction*”[tiab] OR “tooth extraction*”[tiab] OR “teeth extraction*”[tiab] OR “dental extraction*”[tiab]

1.1. HISTORY AND SEARCH DETAILS

Search	Query	Results
#3	#1 AND #2	3,201
#2	"Orthodontics"[Mesh] OR "Orthodont*"[tiab] OR "Dental Marginal Adaptation*"[tiab] OR "Dental internal Adaptation*"[tiab] OR "Dental internal fit*"[tiab] OR "Mandibular Advance*"[tiab] OR "Fixed Functional Appliance*"[tiab] OR "Retainer*"[tiab] OR "Fixed Appliance*"[tiab] OR "Herbst Appliance*"[tiab] OR "Frankel Function*"[tiab] OR "Bimler Appliance*"[tiab] OR "Kinetor*"[tiab] OR "Dental Brace*"[tiab] OR "Clear Aligner*"[tiab] OR "Invisalign"[tiab] OR "transparent Aligner*"[tiab] OR "invisible Aligner*"[tiab] OR "crown lengthen*"[tiab] OR "Occlusal Adjustment*"[tiab] OR "Occlusal Equilibration*"[tiab] OR "Forced Eruption*"[tiab] OR "Tooth Extrusion*"[tiab] OR "Palatal Expansion Techn*"[tiab] OR "Maxillary Expansion*"[tiab] OR "Tooth Movement*"[tiab] OR "Tooth Uprighting*"[tiab] OR "Tooth Intrusion*"[tiab] OR "Tooth Depression*"[tiab] OR "serial extraction*"[tiab] OR "tooth extraction*"[tiab] OR "teeth extraction*"[tiab] OR "dental extraction*"[tiab]	80,678
#1	"Treatment Adherence and Compliance"[Mesh] OR (("patient*"[tiab] OR "Client*"[tiab] OR "Treatment*"[tiab] OR "Therapeutic*"[tiab] OR "medication*"[tiab]) AND ("Complian*"[tiab] OR "Adheren*"[tiab] OR "Noncomplian*"[tiab] OR "Nonadheren*"[tiab] OR "cooperati*"[tiab] OR "noncooperati*"[tiab] OR "acceptance*"[tiab] OR "nonaccept*"[tiab] OR "persistence*"[tiab] OR "nonpersistence*"[tiab] OR "no show*"[tiab]))	529,952
