# Supplementary material for: Patient adherence in orthodontics: a protocol for a scoping review
Source: BDJ Open. 2024 Jul 30;10:62. doi: 10.1038/s41405-024-00249-w (PMC11289492; doi:10.1038/s41405-024-00249-w)
Supplement: Supplementary file 4 — Additional file 3 [file 41405_2024_249_MOESM4_ESM.pdf]

Patient adherence in orthodontics: a protocol for a scoping review

Authors: R.M. van der Bie[1*], A. Bos[1], J. J. M. Bruers[2], R.E.G. Jonkman[1]

First stage screening form

Study's title:		
Authors:		
Do the study's title, abstract or keywords indicate that the study investigates any form of patient adherence in orthodontics?	**If yes, obtain full-text**	**If no, exclude**
Obtain full-text and proceed to second stage screening.	**Yes/No**	

*Correspondence: r.m.vander.bie@acta.nl
R.M. van der Bie, Academisch Centrum Tandheelkunde Amsterdam, Department of Orthodontics, Gustav Mahlerlaan 3004, 1081 LA, Amsterdam, The Netherlands.

Author details

[1]Department of Orthodontics, Academic Centre for Dentistry Amsterdam (ACTA), University of Amsterdam and Vrije Universiteit, Amsterdam, The Netherlands. [2]Department of Oral Public Health, Academic Centre for Dentistry Amsterdam (ACTA), University of Amsterdam and Vrije Universiteit, Amsterdam, The Netherlands.
