# Supplementary material for: Patient adherence in orthodontics: a protocol for a scoping review
Source: BDJ Open. 2024 Jul 30;10:62. doi: 10.1038/s41405-024-00249-w (PMC11289492; doi:10.1038/s41405-024-00249-w)
Supplement: Supplementary file 6 — Additional file 5 [file 41405_2024_249_MOESM6_ESM.pdf]

Patient adherence in orthodontics: a protocol for a scoping review

Authors: R.M. van der Bie[1*], A. Bos[1], J. J. M. Bruers[2], R.E.G. Jonkman[1]

Data charting form

Study's title:	
Authors:	
Year of publication:	
Origin/country of origin:<br>(where the study was published or conducted)	
Aims/purpose:	

Study population and sample size (if applicable):	
Methodology/methods:	
Intervention type/duration, comparater, outcome measures (if applicable):	
Key findings that relate to the research questions:	

*Correspondence: r.m.vander.bie@acta.nl
R.M. van der Bie, Academisch Centrum Tandheelkunde Amsterdam, Department of Orthodontics, Gustav Mahlerlaan 3004, 1081 LA, Amsterdam, The Netherlands.

Author details
[1]Department of Orthodontics, Academic Centre for Dentistry Amsterdam (ACTA), University of Amsterdam and Vrije Universiteit, Amsterdam, The Netherlands. [2]Department of Oral Public Health, Academic Centre for Dentistry Amsterdam (ACTA), University of Amsterdam and Vrije Universiteit, Amsterdam, The Netherlands.
